# Psychometric properties of a Korean version of the Perceived Stress Scale (PSS) in a military sample

**DOI:** 10.1186/s40359-019-0334-8

**Published:** 2019-08-30

**Authors:** Sung Yong Park, Kimberly F. Colvin

**Affiliations:** Department of Educational and Counseling Psychology, University at Albany, State University of New York, ED231, 1400 Washington Avenue, Albany, NY 12222 USA

**Keywords:** Factor structure, Confirmatory factor analysis, Rasch rating scale model, Stress, Young adult

## Abstract

**Background:**

Perceived stress reflects a person’s feeling of how much stress the individual is under at a given time. The Perceived Stress Scale (PSS) is a popular instrument measuring the extent to which individuals perceive situations in their life as excessive relative to the ability to cope. Based on a literature review, however, several issues related to the scale remain: (a) the dimensionality is not established, (b) little information about the individual items exists, and (c) much research is based on university student samples. To address these, this study evaluated the psychometric properties of the Korean version of the Perceived Stress Scale (KPSS) using a military sample.

**Methods:**

This study was conducted in South Korea with 373 military personnel, aged 19–30 years. Both classical test theory (CTT) and the Rasch rating scale model were used to examine the psychometric properties of the KPSS, including factor structure, concurrent validity, reliability, and item analyses.

**Results:**

Internal consistency reliability for the overall and negative/positive perception subscales was.85, .85 and .86, respectively. Based on Rasch reliability, person and item reliability were .82 and .98, respectively. Person and item separation were 2.13 and 7.19, respectively. Concurrent validity was established, with significantly positive association with the measures of depression and negative association with the measure of life satisfaction. Findings from the CFA suggested that a bifactor model with two group factors was the best fit to the observed data. The RSM showed that all but one item had acceptable infit and outfit statistics, and item difficulty ranged from −.73 to 1.22. Besides, the RSM showed positive and moderate inter-item correlations ranging from .42 to .75.

**Conclusions:**

The results provided evidence that a 10-item Korean version of the Perceived Stress Scale was a reliable and valid scale to measure perceived stress in military samples.

**Electronic supplementary material:**

The online version of this article (10.1186/s40359-019-0334-8) contains supplementary material, which is available to authorized users.

## Backgrounds

The Perceived Stress Scale (PSS) is a self-report instrument for measuring the extent to which persons perceive situations in their life as excessively stressful relative to their ability to cope [1]. The PSS was designed for measuring individuals with at least a junior high school education level. It incorporates the theoretical perspective that varying levels of perceived stress can affect the actual experience of stressful events into a widely applicable instrument [1]. Perceived stress has also been linked with coping and perceived ability to cope with stressful events, such that levels of perceived stress are measured relative to a subject’s judgment of own coping ability [1]. Due to its widespread use and discussion in the literature, PSS continues to be utilized and tested for the psychometric properties and validity. The scale allows respondents in secondary school and above to indicate levels of perceived stress as a result of its simple questionnaire format and short, direct questions [2]. The validity and psychometric properties of the Korean version of PSS were examined in the case of military personnel in South Korea.

The PSS was developed to measure global perceived stress experienced outside the bounds of a specific life event and focused on the cognitive appraisal process that includes the appraisal of the stressor and individual’s perceived coping ability [1]. The original PSS included a set of 14 items, consisting of (a) seven items with negative perception of uncontrollability, unpredictability, and inability to cope, and (b) seven items with positive perception of capability to handle stress successfully [1]. This was reduced to 10 items after four were found to exhibit low factor loadings [3]. The PSS has achieved wide acceptance and has been administered to a wide range of study participants. More than 30 language versions of the PSS have been translated and adapted, including Spanish, Portuguese, Mexican Spanish, Chile Spanish, Danish, Norwegian, Swedish, Hebrew, Greek, Italian, German, Moroccan, Bulgarian, Hungarian, Serbian, Korean, Japanese, Mandarin, Taiwanese Mandarin, Thai, Bengali, Malayalam, Tamil, Sinhala, Polish, Lithuanian, Turkish, Russian, Urdu, Arabic, and Finnish [4], and validated on diverse samples, including, for example, university students [1, 5, 6], the general population [3, 7], survivors of suicide [8], adults that participated in a community smoking-cessation program [1], adults with asthma [9], cardiac patients [10, 11], women with breast cancer [12], pregnant and postpartum women [13], teachers [14, 15], workers [14, 16], policewomen [17], and depressed outpatients [18].

**Fig. 1:**
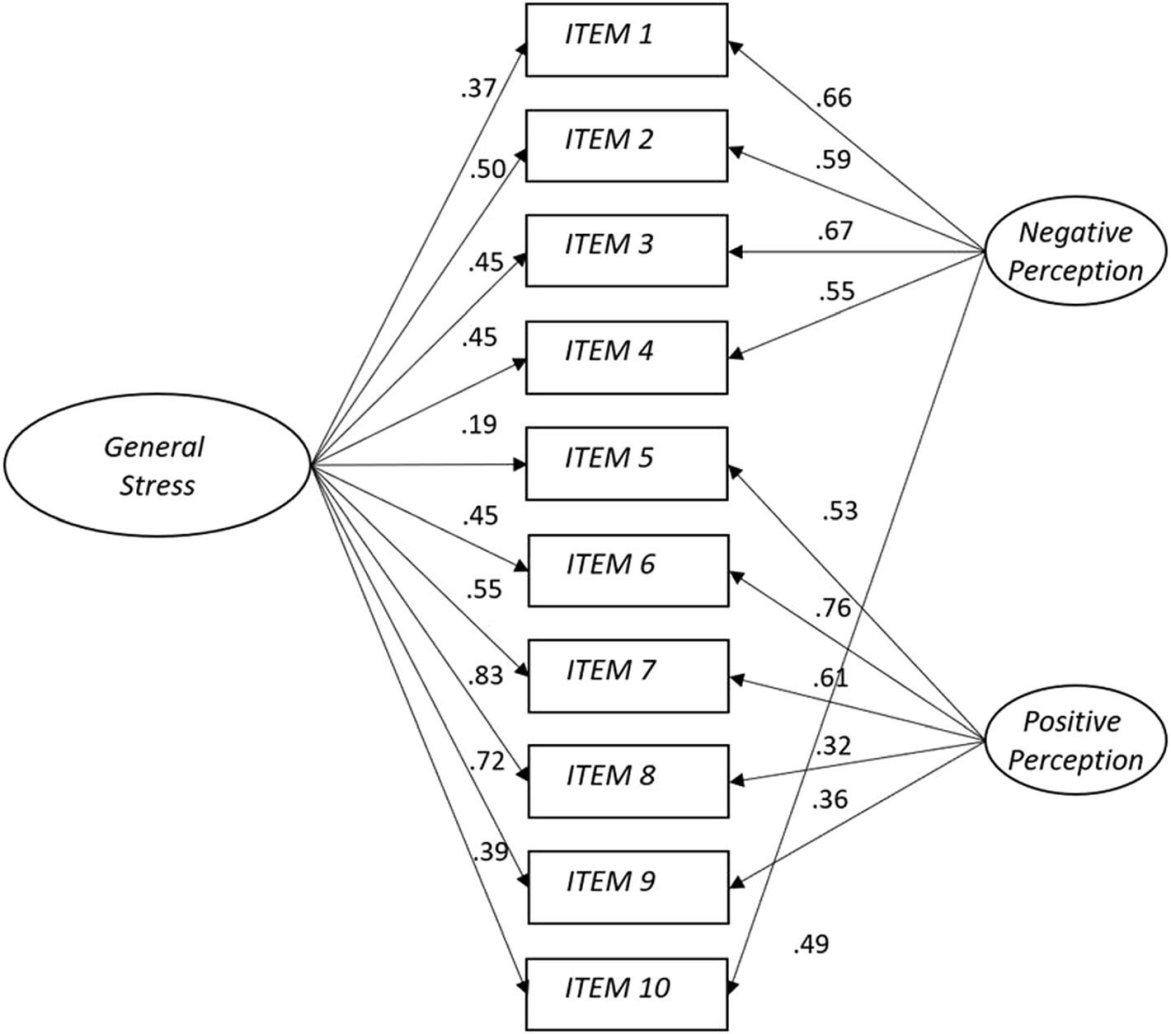
The bifactor model with a general stress factor and two group factors

Much attention has been given to the dimensionality of the PSS. For example, although factor analyses in a study [3] proposed the two-factor model as best fitting the factor structure of the original 14-item PSS and PSS with 10 items, they argued that the distinction between the two factors was irrelevant for purposes of measuring stress. Several following studies have revealed that a two-factor structure ([19, 20]; see [21]) was more acceptable than a one-factor structure for PSS 14 and 10. One study, supported by confirmatory factor analysis (CFA), demonstrated that a second-order factor model was acceptable as an alternative way to use the total score of the two-factor PSS, where “stress” and “counter-stress” are lower-order factors and “perceived stress” is the higher-order factor [12]. The two-factor and second-order factor models do not contain an underlying single construct for stress that explains responses to each of the observed indicators. Recently, a few studies have proposed a bifactor model that addresses these limitations of traditional models used to evaluate the structure of multidimensional constructs [22–25]. As shown in Fig. 1, the bifactor model is different from a second-order model in that subgroup factors are not only included by a general factor underlying all item variables but are also uncorrelated and unique [26].

Even though the PSS has been widely used, there is relatively little in the extant literature about the PSS’s psychometric properties [20], nor about the use of the PSS for a Korean population. To our knowledge, only a few studies translated the original PSS into Korean and evaluated its psychometric properties [27–29]. For example, Park and Seo [29] translated the original 14-item PSS into Korean and evaluated the psychometric properties of the Korean version of PSS (KPSS) with Korean college student samples through both exploratory factor analysis (EFA) and CFA. Their findings revealed that the two-factor structure best fit the data belonging to both positive and negative perception of stress subscales. In addition, as evidence of concurrent validity, negative variables, including depression, anxiety, and negative affect, were positively related to the negative perception factor in the subscales, while the positive perception factor was associated with positive affect.

The PSS measures general stress and is relatively independent of content that is specific to any particular population [1]. Indeed, the PSS has been empirically validated with various populations as described above, but most studies used college students or workers (e.g., professionals and teachers; [21]). Therefore, it is still necessary to validate the PSS with more diverse populations and in various cultures [21]. For example, although several empirical studies revealed that many soldiers are exposed to stress that impacts on mental health conditions [30, 31], no instruments assessing soldiers’ stress levels have been validated in this population. As far as we know, the current study is the first validation study on the PSS for military personnel, in any language. Specifically, South Korean soldiers were and are facing mental and physical health problems, considering the situation in South Korea, where South and North Korea are confronting each other as a divided country, and where the situation changes frequently depending on the interests of the neighboring powers. In addition, given the rigid military culture, soldiers experience difficulties, such as conflicts between ranks, work-related conflicts, and an oppressed group life [30]. Therefore, the Korean military population should be considered distinct from the population of Korean college students who experience stress related to future career plans, intense academic workload and achievement, interpersonal relationships, finance, and personal appearance [32].

The goal of the present study was to examine the psychometric properties of a Korean version of the PSS with 10 items (KPSS10) when administered in a military setting, with a specific interest in the dimensionality of the scale. Using classical test theory (CTT) and factor analysis, we evaluated the factor structure of the scale. To further examine dimensionality, we fit the rating scale model (RSM), a polytomous extension of the Rasch model, to the KPSS10. The Rasch analysis allowed an examination of the performance of individual items on the KPSS10, for which there is little documentation. Then, internal consistency for the items was investigated by both CTT and Rasch reliability statistics. Finally, the concurrent validity of the KPSS10 was examined by comparing scores with those from measures of emotional distress (i.e., depression) and subjective well-being (i.e., life satisfaction).

## Methods

### Participants

At a South Korean military institution, 375 air force soldiers in South Korea, ranging in age from 19 to 30, completed a survey. All participants were male, and the mean length of military service was 17.24 months (SD = 4.17). Regarding the highest level of educational, of the respondents, 5.9% were high school graduates, 84.5% college students, 7.2% college graduates, and 1.9% had attended or completed graduate school. Consent forms and a research description were sent to the air force. After they consented to participate, they completed a paper version of the survey; the survey took approximately 10 min to complete. All but two of the 375 participants who provided complete responses on the KPSS were included in our analyses. Two participants with more than fifteen missing values in responses to all instruments in this survey were excluded from these analyses, yielding a sample size of 373.

In this data set, there were 4 missing values across 10 items and 373 survey respondents, yielding a very low percentage (0.1%) for missing values. Although the Little’s missing completely at random test was significant, it was considered a missing at random pattern based on a visual inspection that showed there are no clusters of missing values. The 4 missing data were imputed using the Expectation-Maximization (EM) algorithm in SPSS Version 24 [33].

The first author conducted the mental health project for Korean military soldiers with a research team; he then obtained the data from a military counselor of the Republic of Korean Air Force (ROKAF) 10th Wing. The current analysis and publication of the data were approved by the ROKAF 10th Wing’s security review.

### Measures

#### Perceived stress scale

The Perceived Stress Scale (PSS; [1]) is a self-report measure consisting of 14 items purported to measure “how unpredictable, uncontrollable, and overloaded respondents find their lives” during the past month [3]. The original version consists of seven negatively stated items and seven positively stated items [1]. Two shortened forms of the PSS 14 were also subsequently developed and validated [3] —the PSS 10 (six negative items and four positive items) and the PSS 4 (two negative items and two positive items). Lee’s review [21] found that the psychometric properties of the PSS 10 were more effective in measuring the perceived stress than those of the PSS 14 and 4 items.

The Korean version translated and evaluated by Park and Seo [29] is made up of five negatively stated items (i.e., 1, 2, 3, 11, and 14 in the original PSS 14) and five positively stated items (i.e., 4, 5, 6, 7, and 10 in the original version) depending on factor loadings over 0.5 among the full 14 items. Participants indicate their response to the KPSS using a 5-point Likert-type scale ranging from 0 (never) to 4 (very often). To produce the total score, the five positively stated items in questionnaires were reversed, thus, higher scores indicate higher perceived stress. For the current items used in the study see the Additional file 1. Park and Seo [29] found that a two-factor solution, with positive and negative perception as the subfactors, was supported (α = .74 for positive perception and .77 for negative perception). Concurrent validity was established by moderate correlations with depression, anxiety, negative affect, and positive affect.

#### Center for epidemiologic studies depression scale

There is a growing body of evidence identifying the stress-depression connection (see [21]). To establish concurrent validity, a comparison was made with the CES-D, a self-report scale designed to measure the current level of depressive symptoms for general population [34]. The scale consists of 20 items using a 4-point scale ranging from 0 (*Rarely or none of the time, less than 1 day*) to 3 (*Most or all of the time, 5–7 days*). For example, item 1 is “I was bothered by things that usually don’t bother me.” The CES-D has four subfactors: depressive affect, positive affect, somatic symptoms, and interpersonal difficulties [34]. We used the Korean version of the CES-D translated and validated by Chon, Choi, and Yang [35], which demonstrated the same factor structure with the original CES-D and high internal consistency (α = .91). The internal consistency reliability estimate in the present study was .90.

#### Satisfaction with life scale

As previous literature suggested that perceived stress was predictive of low levels of life satisfaction [36], the Satisfaction with Life Scale (SWLS; [37]) was also administered to assess concurrent validity. The SWLS was designed to assess cognitive judgments of life satisfaction using a short instrument with only five items. The responses to each item (e.g., “So far I have gotten the important things I want in life”) range from 1 (strongly disagree) to 7 (strongly agree), where higher scores indicate higher levels of life satisfaction. We used the Korean version of the SWLS, which has been translated and evaluated for psychometric properties in a Korean population [38]. In Kim’s study [38], the Cronbach’s alpha was .84, and the current sample yielded the alpha coefficients of .86.

### Data analysis

Both CTT and Rasch RSM were used to evaluate the psychometric properties of the KPSS10, including factor structure, concurrent validity, reliability, and item analyses. Reliability of the KPSS10 was reported in two ways using Cronbach’s alpha and item-total correlation. In general, a Cronbach’s alpha value of 0.70 is recommended as a minimum acceptable criterion for internal consistency [39]. Furthermore, Rasch-based person and item reliability and separation were reported. The person reliability index refers to the expected replicability of person placement if this sample was given other items measuring the same construct, while the item reliability index indicates the replicability of item placements resulting from other samples who behaved in the same way [40]. Both reliability indices range from 0 to 1, with values greater than .90 for items and .80 for persons being regarded as acceptable [40]. The separation index indicates an estimate of the spread or separation of items or persons along the measured variable, with adequate separation in persons or items values of at least 2.0 regarded as acceptable [40]. Concurrent validity was investigated by evaluating the correlational relationship with measures of negative emotion (e.g., depression), using the CES-D and subjective well-being (e.g., satisfaction with life), using the SWLS. We expected the KPSS10 to correlate positively with the CES-D and to correlate negatively with the SWLS.

We used CFA to examine the dimensionality of the KPSS10. Based on the factor structures reported in the PSS literature, four different factor configurations of the KPSS10 were extracted: (a) a single-factor unidimensional model that all 10 items are assumed to measure a single stress factor [8], (b) a two-factor model with two covariate factors [19–21, 27, 29], (c) a bifactor model with a general stress factor and a nuisance factor consisting of the five reversed items [23], and (d) a bifactor model with a general stress factor accounting for the commonality shared by the items and two subfactors reflecting the unique variance not accounted for by the general stress factor, as seen in Fig. 1 [22, 24, 25]. The bifactor model allowed us to test whether the KPSS10 was a general measure of perceived stress with another specific underlying dimension.

To examine the adequacy of model-fit, we reported the comparative fit index (CFI) representing incremental fit, standardized root-mean-square residual (SRMR) for absolute fit, and root-mean-square error of approximation (RMSEA) identifying parsimonious fit. In our data, Mardia’s multivariate kurtosis coefficient of 17.40 indicated the absence of multivariate normality [41]. Given this result and the ordinal nature (a five-point Likert-type scale) of the KPSS, robust maximum likelihood estimation was used in the CFA analyses in EQS 6.1 [42], instead of using maximum likelihood estimator.

Next, as an indicator of unidimensionality used in a bifactor model, we computed the explained common variance (ECV) that is a ratio of common variance attributable to the general factor (ECV; [43]). High ECV values indicate data that have a strong general factor compared to other specific group factors; when values are greater than .70, the common variance can be considered as unidimensional [43].

To further explore dimensionality and assess the relative location of items and respondents, we used WINSTEPS version 4.01 [44] to fit the rating scale model (RSM; [40, 45]) to our data, while accounting for the dimensionality as found in the factor analyses. Contrary to CTT, Rasch analyses enable researchers to analyze the properties of items, such as item difficulty and item discrimination. The RSM is an extension of the Rasch model for polytomous data [45, 46]. The RSM estimates the location of the respondents and the KPSS10 items on the same scale, in this case, the scale of perceived stress. The RSM manipulates only one set of threshold parameters of across all items on the scale, indicating a common rating scale structure for all items [40]. For each item, the overall location of the item is estimated, along with the location of the thresholds, that is the location on the scale where the likelihood of a response in a particular category changes. In other words, the scale is divided into sections based on the most likely response. Therefore, the RSM is suitable when one expects that psychological distances between categories are the same across all items [47].

However, to conduct the Rasch analysis, we had two choices: the RSM and the partial credit model (PCM). While the PCM allows for the item response categories to differ across items, in the case of Likert-type items a strong case needs to be made to use the PCM over the RSM [48]. Theoretically, we would argue that because respondents were presented with the same response options across all items, the set of responses should be treated the same across all items. However, because it is possible that there was an interaction between the respondents and the items leading to a discrepant use of response categories across items, we initially fit both the RSM and PCM. The ordering and spacing of the thresholds remained roughly the same across all items in both the PCM and the RSM, indicating that the data would support the selection of the RSM. We next compared the person and item reliability index obtained from the two models. The person reliability is .85 for the PCM and .82 for the RSM, and the item reliability is .98 for both PCM and RSM. Given the similarity of threshold spacing, fit indices, and the theoretical argument that the set of response categories is the same across items, we decided to fit the more parsimonious RSM, rather than the PCM.

Finally, after fitting the RSM we used WINSTEPS to conduct a principal components analysis of the standardized residuals [49]. If the underlying factor fit by the RSM accounts for most of the variance in the original data, then it is expected that the resulting components of residuals will represent noise. The results of the analysis can be used to separate items into groups to determine if some of the unaccounted variance (variance not accounted for in the RSM) can be explained by an additional factor or factors.

## Results

### Reliability

As shown in Table 1, Cronbach’s alpha coefficients indicated good internal consistency for the overall KPSS10 (α = .85), for the negative perception subscale (α = .85), and for the positive perception subscale (α = .86) [40]. Cronbach’s alpha if item deleted for all ten items ranged from .83 to .87. Item 5 was the only item that would yield a slightly higher alpha if removed. Item-total correlations for individual items and each factor were also investigated, and ranged from .45 to .75, showing over the generally adopted cutoff criteria (>.40; [50]). Therefore, all items appeared worthy of retention. These two types of statistics on internal consistency reliability indicate that the KPSS10 contains items that are particularly intercorrelated. Regarding the results from Rasch-based reliability, both person and item reliability indices were acceptable: .82 and .98, respectively. In addition, results pertaining to person and item separation were 2.13 and 7.16, respectively. In general, these reliability results indicate good separation in the KPSS10 for both persons and items [40].

**Table 1:** Descriptive Statistics and Correlations of Measures

Measure	1	2	3	4	5	M	SD	α
1. KPSS Total	1					2.27	.57	.85
2. KPSS Negative perception	.84	1				2.16	.71	.85
3. KPSS Positive perception	.81	.36	1			2.38	.66	.86
4. CES-D	.62	.56	.45	1		.52	.41	.90
5. Life Satisfaction	−.49	−.42	−.38	−.47	1	4.27	1.19	.86

Note. *N* = 373. All correlation coefficients are significant at *p* < .01; KPSS = Korean version of the Perceived Stress Scale with 10 items; KPSS negative perception indicates the negatively worded items, and KPSS positive perception means the positively worded items; The KPSS positive items were reverse-coded

### Concurrent validity

As expected, we found statistically significant positive associations between the KPSS total scores and two subscale scores and depression: CES-D (*r* = .61, .56, and 44, respectively, *p* < .01), as well as a negative association with life satisfaction: SWLS (*r* = −.48, −.42, and − .37, respectively, *p* < .01). All correlation coefficients ranged between .37 and .61, which are considered to be medium or strong correlations [51]. In sum, these correlations provide evidence of concurrent validity for the KPSS10 (see Table 1).

### Confirmatory factor analysis (CFA)

Results from the CFA supported a bifactor model for the KPSS10. Fit indices mentioned above for the factor structure including one-factor, two-factor, and bifactor models are provided in Table 2.

**Table 2:** Confirmatory Factor Analyses of the KPSS

Model	S-B χ^2^	*df*	CFI	SRMR	RMSEA [90% CI]
One-factor model	480.914	35	.649	.157	.185 [.170, .200]
Two-factor model	117.885	34	.934	.063	.081 [.065, .097]
Bifactor model (A)	82.469	30	.959	.033	.069 [.051, .086]
Bifactor model (B)	52.051	25	.979	.039	.054 [.033, .074]

Note. *CFI* Comparative fit index, *SRMR* standardized root-mean-square residual, *RMSEA* room-mean-square error of approximation, *CI* confidence interval; the bifactor model (A) includes a general stress factor and a nuisance factor, while the bifactor model (B) consists of a general stress factor and two group factors

The initial one-factor CFA model had poor model fit using Hu and Bentler’s joint criteria [52]. Although the two-factor model yielded an acceptable fit to the data, the bifactor model (A) with the general stress factor and one nuisance factor demonstrated better fit as compared to the two-factor model, ∆*S-B* χ^2^ (4) = 35.416, *p* < .001. All factor loadings were significant for the general and the nuisance factor except for item 5. Considering this, we tried to conduct the second bifactor model (B) in which all 10 items load onto the general stress factor as well as on the two group factors. The bifactor model (B) yielded better fit, *S-B* χ^2^ (25) = 52.051, *p* < .001, CFI = .979, SRMR = .039, RMSEA = .054 [.033, .074], and shown a significant improvement in fit indices, as compared to the first bifactor model (A), ∆*S-B* χ^2^ (5) = 30.418, *p* < .001. In contrast to the bifactor model (A), all factor loadings were significant for the general and the two group factors (all *ps* < .001), as shown in Fig. 1. Our findings supported the bifactor model with the general stress factor and the two group factors labeled as “negative perception and positive perception” as the best fitting model.

The ECV in our supported model was .45, indicating that the general stress factor accounted for almost half the common variance. Because the bifactor model (B) yielded the best fit and the two group factors related to the positive or negative wording of the item, we conducted Rasch analyses focusing on the KPSS10 as a whole in a confirmatory manner, rather than on the two subscales. The two group factors could be considered as superficial and not meaningful [3] because they represented the direction of the wording of the items rather than the content of the item; in addition, most research and clinical contexts generally use a single summed PSS score. Reckase [53] argued that item estimates are defensible when the first component of principal components analysis accounts for at least 20% of the variance; in our data the first component accounted for 44% of the variance. To further confirm that a Rasch analysis on all ten items at once was appropriate, we compared the relative item positions and person estimates from an RSM analysis of all ten items with those from analyses of the positive and negative items separately. The person estimates from an RSM analysis with only the positive items correlated .92 with the person estimates based on all ten items, while the estimates based on the negative items correlated .73 with the estimates based on all ten items. The relative positioning of the items when calibrated separately as positive and negative items were the same as when all ten items were calibrated simultaneously. These results, coupled with the fact that the first eigenvalue accounts for 44% of the variance, well over the minimum recommended of 20%, indicated that a single RSM analysis of all ten items was appropriate to generate item and person estimates.

### Rasch rating scale model

The RSM was fit to the data to evaluate item performance of the KPSS10 with the military sample of respondents based on item difficulty, separation index, item misfit detection, item discrimination, and Pearson point measure correlation (PTMEA). The results are provided in Table 3. Ten items are arranged in item difficulty values, from most difficult item to respond to at the top (item 3), to the least difficult item to respond to at the bottom (item 5). For instance, the item 3 “Cannot overcome pilling up difficulties” was more difficult to endorse, referring to higher stress severity, whereas item 5 “Dealing successfully with day-to-day problems and annoyances” was the most likely to obtain a response of “never,” meaning lower stress severity. In addition, the item separation index of 7.19 is also a good separation in the KPSS items and indicates that these items define adequately a distinct hierarchy of item difficulty [54].

**Table 3:** Rasch Rating Scale Model (RSM) Analyses

KPSS Item		Difficulty	Estimated Discrimination	Infit MNSQ	Outfit MNSQ	PTMEA
Item 3 (14)	Cannot overcome mounting difficulties	1.22	1.23	0.82	0.80	0.66
Item 1 (2)	Unable to control the important things	1.03	.93	1.10	1.14	0.60
Item 6 (5)	Effectively cope with important changes in your life	0.02	1.21	0.82	0.81	0.66
Item 7 (6)	Confident about your ability to handle your problems	0.02	1.18	0.84	0.83	0.68
Item 9 (10)	Feel that you are on top of things	−0.17	1.33	0.70	0.69	0.70
Item 4 (1)	Upset because of something that happened unexpectedly	−0.23	.79	1.21	1.22	0.64
Item 2 (3)	Feel nervous or stressed	−0.24	.84	1.16	1.14	0.68
Item 8 (7)	Feel that things are going your way	−0.45	1.38	0.63	0.64	0.75
Item 10 (11)	Feel angry because of things that happened that are outside of your control	−0.48	.82	1.15	1.17	0.59
Item 5 (4)	Deal successfully with day-to-day problems and annoyances	−0.73	.40	**1.58**	**1.66**	0.42

Note. KPSS10 is Korean version of the Perceived Stress Scale 10 items; numbers in parentheses refer to the original number of the PSS-14 [1]; difficulty means perceived stress severity level; infit/outfit statistics in bold are larger than 1.4 and indicate misfit; PTMEA = the point-measure correlation

**Fig. 2:**
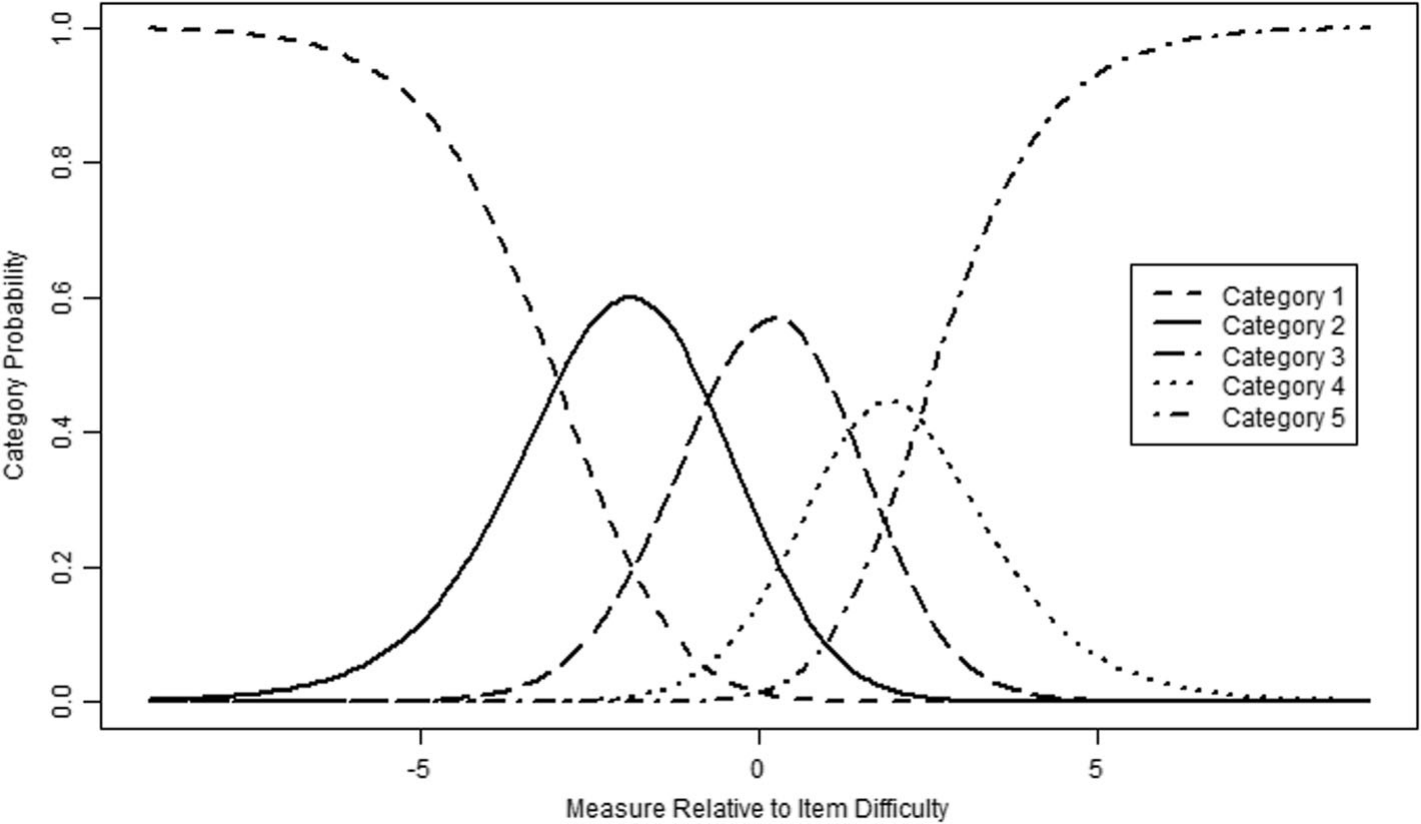
The relative category probability curves for items of the KPSS10

Next, item misfit was evaluated using the following Rasch fit indicators. Mean-square fit statistics (MNSQ) were examined; specifically, infit (weighted mean square) and outfit (unweighted mean square) determine how well each item contributes to defining one common construct. In the case of a Likert scale, the expected MNSQ value is 1.0, infit and outfit values from 0.6 to 1.4 are within acceptable bounds for Likert scale measurements, indicating construct homogeneity with other items in a scale [47, 55]. MNSQ values greater than 1.4 may indicate a lack of construct homogeneity with other items in a scale, while values less than 0.6 may indicate item redundancy [47, 55]. As shown in the Table 3, all items of the KPSS10 had acceptable infit and outfit statistics between 0.60 and 1.40, except for only one item (item 5) revealing both infit and outfit statistics larger than 1.4. Moreover, most items on the KPSS10 had positive, moderate, inter-item correlations ranging from .42 to .75, indicating that all items on the KPSS10 function as intended (see the PTMEA in Table 3; [54]). Although Rasch models are assumed that all item discriminations are regarded as equal, empirical item discriminations are never equal so that WINSTEPS produces item discrimination estimates post-hoc [54]. The estimates of the item discrimination distributed all around from .40 (item 5) to 1.38 (item 8), including five under-discriminating items and five over-discriminating items shown in Table 3. Finally, the Probability Curves revealed that the 5-point Likert-type scale in the KPSS10 were ordered as expected, indicating that the differentiation of each category along the attribute measurement was verified (see Fig. 2).

Finally, the principal components analysis of the standardized residuals revealed that of the unexplained variance 35% was attributable to the first component, indicating that the component is accounting for more than just noise. In fact, the first component separated the 10 items into two distinct groups: the five items with positive wording and the five items with negative wording. The remaining components accounted for roughly equal variance, indicating no additional conceptual dimensions to the data.

### Appropriateness of item difficulty for military samples

**Fig. 3:**
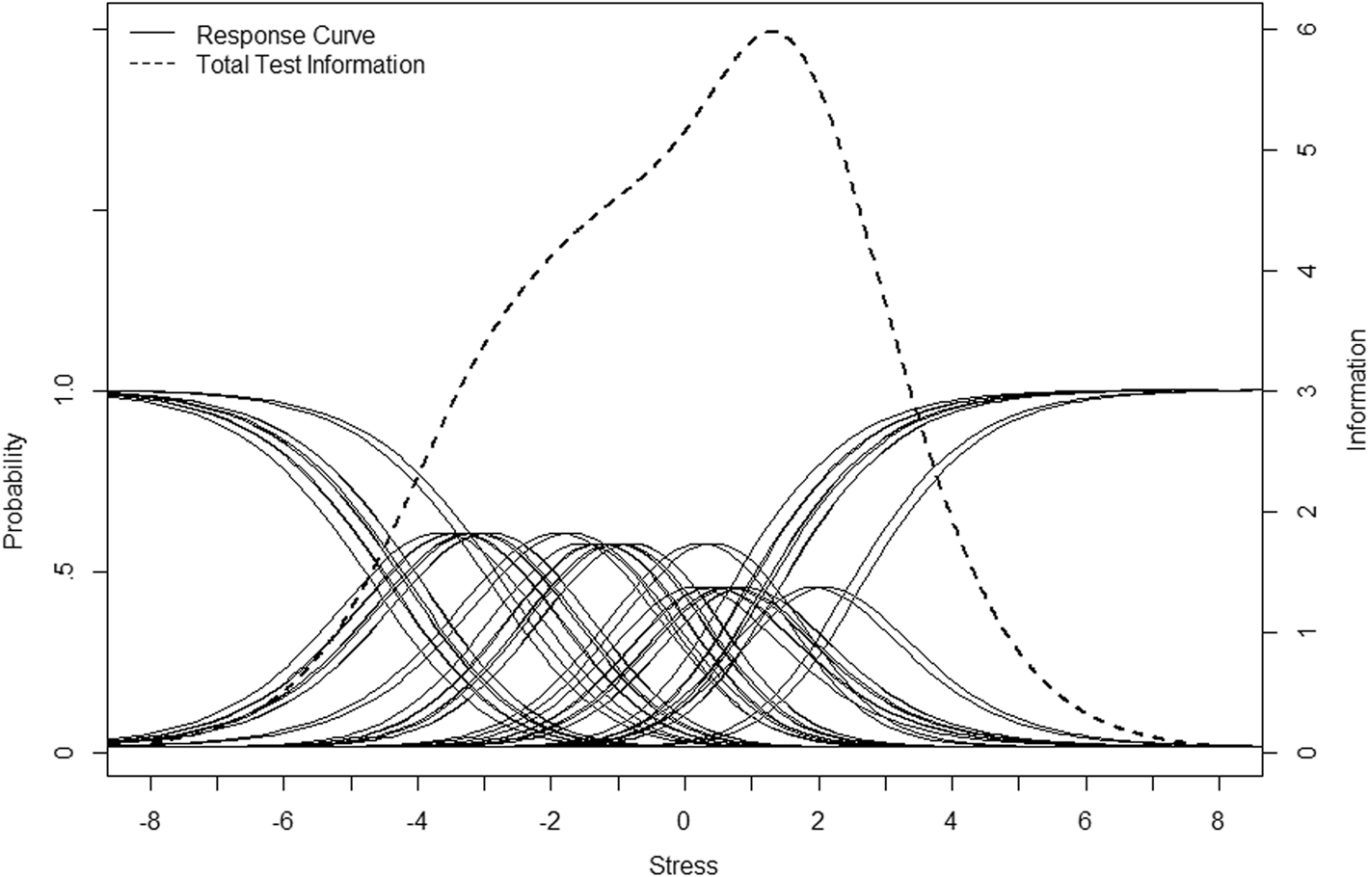
Items’ category probability curves and the total test information curve

Because the Rasch model estimates person and item locations on the same scale, we can investigate whether the item difficulty level of the KPSS10 is appropriate for the current sample. If the KPSS-10 was appropriately targeted for the level of the sample being tested, there should be considerable overlap between the range of the person trait measures and the total test information curve and some of the item category probability curves. As shown in Fig. 3, the test information curve and the items, depicted by each item’s individual category probability curves, were aligned with most of the current sample’s locations along the stress scale (*M* = − 1.45, *SD* = 1.46, *minimum* = − 6.60, *maximum* = 2.99). The one exception is for the few people with the lowest estimate of stress, − 6.60, where the items were not targeted to the low end of the stress scale. This means the KPSS10 items could measure a more severe level of perceived stress than was needed for this nonclinical sample of South Korean soldiers, but still more than adequately targeted almost the entire sample.

## Discussion

In this study, we investigated the psychometric properties of the Korean version of the Perceived Stress Scales in a sample of military personnel in South Korea, using the KPSS 10 items translated and validated by Park and Seo [29]. Overall, both CTT analyses and Rasch modeling provided evidence that the KPSS10 is a reliable and valid instrument measuring perceived stress within military samples in South Korea.

The CFA analyses to compare four competing models’ goodness-of-fit demonstrated that a bifactor model with a general stress factor and two group factors was the best fit to our data. Regarding two group factors, our model was more consistent with the bifactor model supported by previous studies [22, 25], rather than Perera et al.’s [23] model with only one nuisance factor consisting of four negatively worded items. In addition to the general stress factor reflecting the overlap across all items, two group factors in our findings indicate that the five negatively worded items of the KPSS10 were loaded onto the negative perception factor and the positively worded remaining five items were loaded onto the positive perception factor. It is worthy of note that when all the items’ loadings on the general factor will be stronger than those on the group factors, a bifactor structure could be viewed as mostly unidimensional. This underlying hypothesis was not supported by factor loadings in our bifactor model; items loaded more strongly on the group factors than on the general stress factor. The principal components analysis on the residuals from the RSM analysis demonstrated the same underlying factor structure as the CFA: one general stress factor with the unexplained variance dividing the items into the positive and negatively worded items.

Regarding the reliability, the overall and two subscales’ Cronbach’s alpha coefficients (.85, .85, and .86, respectively) indicate that the KPSS10 had a good internal consistency reliability for the Korean military sample. Our findings were higher than those observed in the original study [3]. Concurrent validity of the full and the subscales of the KPSS was established, with significantly positive associations with the measures of depression and negative association with the measure of life satisfaction. In other words, high KPSS10 scores were correlated with reports of increased depression and dissatisfaction. These findings were consistent with the prior findings showing significant correlations with measures of distress and subjective well-being constructs [3, 22, 56]. Contrary to the earlier findings, however, the two subscales correlated positively with each other. This finding was consistent with the validation study based on Korean college students [29].

To our knowledge, this is the first study to use the Rasch RSM to investigate the PSS. Our findings were indicated by the adequate MNSQ fit of almost items, evenly separated item difficulty, acceptable discrimination, and fairly strong positive PTMEA correlations. According to the results showing good separation in the KPSS10 for both persons and items, the KPSS10 may be sensitive enough to discriminate between high and low stressed respondents [54]. The majority of the respondents’ scale locations overlapped with the item category probability curves in the middle and at the lower end of the scale. Given that the PSS was designed to measure the degree to which individuals perceive their lives as stressful in both clinical and non-clinical population [1], this finding can be regarded as reasonable, concluding that the KPSS10 items are designed to measure more severe levels of perceived stress than was observed in our non-clinical sample of soldiers.

There are some limitations to be considered in interpreting the findings. First, the KPSS10 [29] that we used in this study, is a translated and validated version that is adapted for the Korean population. In this process, the KPSS10 included two items not present in the original English PSS10 [3] so that it will be somewhat difficult to compare directly with other previous findings. Second, considering all the items and all subfactors, positive correlations were found, justifying computing a total score of the KPSS10. Another limitation of our study is that is we could not compare KPSS10 scores to another measure of stress to assess convergent validity, instead, we established concurrent validity with expected significant correlations among the mental health measures in this study. Finally, it may be difficult to generalize from our findings, because of our particular sample. The military sample in the study was not representative of the military population in other countries because of the nature of military service in South Korea, in which participation is mandatory. The KPSS10 was also only administered at one-time point, and the sample only included males, therefore, future studies will have to assess test-retest reliability and include women in the study sample.

## Conclusions

In a South Korean military sample, the Korean version of the PSS proved to be a reliable instrument with concurrent validity. We found evidence that while a bifactor model best fit the data, the data are unidimensional enough to conduct a Rasch analysis. To our knowledge, this is the first study to use the Rasch rating scale model to investigate the PSS. The results indicated a good separation in the KPSS for both persons and items, demonstrated that the KPSS is sensitive enough to discriminate between high and low stressed respondents. Given that the PSS was designed to measure the degree to which individuals perceive their lives as stressful in both clinical and non-clinical populations, it is not surprising that we found the Korean version of the PSS to be an adequate measure of perceived stress in our non-clinical sample of soldiers.

## Additional file

Additional file 1: Korean Version of the Perceived Stress Scale (KPSS). (PDF 168 kb)

## Data Availability

The dataset analyzed during the current study is not publicly available because the data are controlled by the Republic of Korea Air Force 10th Fighter Wing but are available from the corresponding author on reasonable request.

## Abbreviations

CES- D: Center for Epidemiological Studies
CFI: Comparative fit index
CTT: Classical test theory
ECV: Explained common variance
KPSS: Korean version of Perceived Stress Scale
MNSQ: Mean-square fit statistics
PSS: Perceived Stress Scale
PTMEA: Pearson point measure correlation
RMSEA: Root-mean-square error of approximation
RSM: Rating scale model
SRMR: Standardized root-mean-square residual
SWLS: Satisfaction with Life Scale

## References

[1] Cohen S, Kamarck T, Mermelstein R (1983). A global measure of perceived stress. J Health Soc Behav.

[2] Karam F, Bérard A, Sheehy O, Huneau MC, Briggs G, Chambers C, Einarson A, Johnson D, Kao K, Koren G, Martin B (2012). Reliability and validity of the 4-item perceived stress scale among pregnant women: results from the OTIS antidepressants study. Res Nurs Health.

[3] Cohen S, Williamson GM, Spacapan S, Oskamp S (1988). Perceived stress in a probability sample of the United States. The social psychology of health: Claremont symposium on applied social psychology.

[4] Cohen’s laboratory for the Study of Stress, Immunity, and Disease. Dr. Cohen’s Scales. 2018. http://www.psy.cmu.edu/~scohen/index.html. Accessed 15 Jun 2018.

[5] Örücü MÇ, Demir A (2009). Psychometric evaluation of perceived stress scale for Turkish university students. Stress Health.

[6] Roberti JW, Harrington LN, Storch EA (2006). Further psychometric support for the 10-item version of the perceived stress scale. J Coll Couns.

[7] Andreou E, Alexopoulos EC, Lionis C, Varvogli L, Gnardellis C, Chrousos GP, Darviri C (2011). Perceived stress scale: reliability and validity study in Greece. Int J Environ Res Public Health.

[8] Mitchell AM, Crane PA, Kim Y (2008). Perceived stress in survivors of suicide: psychometric properties of the perceived stress scale. Res Nurs Health..

[9] Sharp LK, Kimmel LG, Kee R, Saltoun C, Chang CH (2007). Assessing the perceived stress scale for African American adults with asthma and low literacy. J Asthma.

[10] Leung DY, Lam TH, Chan SS (2010). Three versions of perceived stress scale: validation in a sample of Chinese cardiac patients who smoke. BMC Public Health.

[11] Pbert L, Doerfler LA, DeCosimo D (1992). An evaluation of the perceived stress scale in two clinical populations. J Psychopathol Behav Assess.

[12] Golden-Kreutz DM, Browne MW, Frierson GM, Andersen BL (2004). Assessing stress in cancer patients: a second-order factor analysis model for the perceived stress scale. Assessment..

[13] Chaaya M, Osman H, Naassan G, Mahfoud Z (2010). Validation of the Arabic version of the Cohen perceived stress scale (PSS-10) among pregnant and postpartum women. BMC Psychiatry..

[14] Almadi T, Cathers I, Mansour AM, Chow CM (2012). An Arabic version of the perceived stress scale: translation and validation study. Int J Nurs Stud.

[15] Reis RS, Hino AA, Rodriguez Añez CR (2010). Perceived stress scale: reliability and validity study in Brazil. J Health Psychol.

[16] Lesage FX, Berjot S, Deschamps F (2012). Psychometric properties of the French versions of the perceived stress scale. Int J Occup Med Environ Health.

[17] Wang Z, Chen J, Boyd JE, Zhang H, Jia X, Qiu J, Xiao Z (2011). Psychometric properties of the Chinese version of the perceived stress scale in policewomen. PLoS One.

[18] Wongpakaran N, Wongpakaran T (2010). The Thai version of the PSS-10: an investigation of its psychometric properties. Biopsychosoc Med.

[19] Hewitt PL, Flett GL, Mosher SW (1992). The perceived stress scale: factor structure and relation to depression symptoms in a psychiatric sample. J Psychopathol Behav Assess.

[20] Taylor JM (2015). Psychometric analysis of the ten-item perceived stress scale. Psychol Assess.

[21] Lee EH (2012). Review of the psychometric evidence of the perceived stress scale. Asian Nurs Res.

[22] Jovanović V, Gavrilov-Jerković V (2015). More than a (negative) feeling: validity of the perceived stress scale in Serbian clinical and non-clinical samples. Psihologija..

[23] Perera MJ, Brintz CE, Birnbaum-Weitzman O, Penedo FJ, Gallo LC, Gonzalez P, Gouskova N, Isasi CR, Navas-Nacher EL, Perreira KM, Roesch SC (2017). Factor structure of the perceived stress Scale-10 (PSS) across English and Spanish language responders in the HCHS/SOL sociocultural ancillary study. Psychol Assess.

[24] Reis D, Lehr D, Heber E, Ebert DD. The German version of the Perceived Stress Scale (PSS-10): evaluation of dimensionality, validity, and measurement invariance with exploratory and confirmatory bifactor modeling. Assessment. 2017; doi:1073191117715731. 10.1177/1073191117715731 28627220

[25] Wu SM, Amtmann D (2013). Psychometric evaluation of the perceived stress scale in multiple sclerosis. ISRN Rehabil.

[26] Gustafsson JE, Balke G (1993). General and specific abilities as predictors of school achievement. Multivariate Behav Res.

[27] Lee EH, Chung BY, Suh CH, Jung JY (2015). Korean version of the perceived stress scale (PSS-14, 10 and 4): psychometric evaluation in patients with chronic disease. Scand J Caring Sci.

[28] Hong Gwi-Ryung Son, Kang Hye-Kyung, Oh Eunmi, Park YoungOk, Kim Haesook (2015). Reliability and Validity of the Korean Version of the Perceived Stress Scale-10 (K-PSS-10) in Older Adults. Research in Gerontological Nursing.

[29] Park JO, Seo YS (2010). Validation of the perceived stress scale (PSS) on samples of Korean university students. Korean J Psychol.

[30] Koo SS (2006). A study on mental health of new generation soldiers. Mental Health Soc Work.

[31] Martin PD, Williamson DA, Alfonso AJ, Ryan DH (2006). Psychological adjustment during army basic training. Mil Med.

[32] Lee DH, Kang S, Yum S (2005). A qualitative assessment of personal and academic stressors among Korean college students: an exploratory study. Coll Stud J.

[33] IBM Corp (2016). Released. IBM SPSS statistics for windows, version 24.0.

[34] Radloff LS (1977). The CES-D scale: a self-report depression scale for research in the general population. Appl Psychol Meas.

[35] Chon KK, Choi SC, Yang BC (2001). Integrated adaptation of CES-D in Korea. Korean J Health Psychol.

[36] Abolghasemi A, Varaniyab ST (2010). Resilience and perceived stress: predictors of life satisfaction in the students of success and failure. Procedia Soc Behav Sci.

[37] Diener E, Emmons RA, Larsen RJ, Griffin S (1985). The satisfaction with life scale. J Pers Assess.

[38] Kim JH (2007). The relationship between life satisfaction/life satisfaction expectancy and stress/well-being: an application of motivational states theory. Korean J Health Psychol.

[39] Kline P (2000). A psychometrics primer.

[40] Wright BD, Masters GN. Rating scale analysis: Rasch measurement. Chicago: Mesa Press; 1982.

[41] Mardia KV (1970). Measures of multivariate skewness and kurtosis with applications. Biometrika..

[42] Bentler PM, Wu EJ. EQS 6.1 for Windows: Users' guide. Encino: Mulivariate Software; 2003.

[43] Rodriguez A, Reise SP, Haviland MG (2016). Applying bifactor statistical indices in the evaluation of psychological measures. J Pers Assess.

[44] Linacre JM. Winsteps® Rasch measurement computer program, Version 4.1. Beaverton: Winsteps.com; 2017.

[45] Andrich D (1988). Rasch models for measurement.

[46] Embretson SE, Reise SP (2000). Item response theory for psychologists.

[47] Bond TG, Fox CM (2015). Applying the Rasch model: fundamental measurement in the human sciences.

[48] Wright BD (1998). Model selection: rating scale model (RSM) or partial credit model (PCM)?. Rasch Meas Trans.

[49] Linacre JM (1998). Detecting multidimensionality: which residual data-type works best?. J Outcome Meas.

[50] Ware JE, Gandek B (1998). Methods for testing data quality, scaling assumptions, and reliability: the IQOLA project approach. J Clin Epidemiol.

[51] Cohen J (1988). Statistical power analysis for the behavioral sciences.

[52] Hu LT, Bentler PM (1999). Cutoff criteria for fit indexes in covariance structure analysis: conventional criteria versus new alternatives. Struct Equ Modeling.

[53] Reckase MD (1979). Unifactor latent trait models applied to multifactor tests: results and implications. J Edu Stat.

[54] Linacre JM (2005). A user’s guide to WINSTEPS.

[55] Wright BD, Linacre JM, Gustafson JE, Martin-Lof P (1994). Reasonable mean-square fit values. Rasch Meas Trans.

[56] Klein EM, Brähler E, Dreier M, Reinecke L, Müller KW, Schmutzer G, Wölfling K, Beutel ME (2016). The German version of the perceived stress scale–psychometric characteristics in a representative German community sample. BMC Psychiatry.

