# Supplementary material for: Psychometric properties of a Korean version of the Perceived Stress Scale (PSS) in a military sample
Source: BMC Psychol. 2019 Aug 30;7:58. doi: 10.1186/s40359-019-0334-8 (PMC6716865; doi:10.1186/s40359-019-0334-8)
Supplement: Supplementary file 1 — Korean Version of the Perceived Stress Scale (KPSS). (PDF 168 kb) [file 40359_2019_334_MOESM1_ESM.pdf]

Korean Version of Perceived Stress Scale (KPSS)

이 척도는 지난 한 달 동안 어떤 감정과 생각이 들었는지 물어보는 것입니다. 각 질문에 대해 당신이 얼마나 자주 느끼거나 생각했는지를 "V" 표시해 주시기 바랍니다.

(The questions in this scale ask you about your feelings and thoughts **during the last month**. In each case, you will be asked to indicate by circling *how often* you felt or thought a certain way)

KPSS Item #(PSS)	KPSS 10	Never				Very often
1(2)	나는 생활 속에서 중요한 일들을 통제할 수 없다고 느꼈다. (Unable to control the important things in your life)	0	1	2	3	4
2(3)	나는 초조하거나 스트레스가 쌓인다고 느꼈다. (Nervous or stressed)	0	1	2	3	4
3(14)	나는 어려운 일이 너무 많이 쌓여서 극복할 수 없을 것처럼 느껴졌다. (Difficulties are piling up so high that you cannot overcome them)	0	1	2	3	4
4(1)	나는 예상치 못한 일이 생겨서 기분이 나빠졌다. (Upset because of something that happened unexpectedly)	0	1	2	3	4
5(4)	나는 짜증나고 성가신 일들을 성공적으로 처리했다. (Dealing successfully with day-to day problems and annoyances)	0	1	2	3	4
6(5)	나는 생활 속에서 일어난 중요한 변화들에 대해 효과적으로 대처했다. (Effectively coping with important changes that are occurring in your life)	0	1	2	3	4
7(6)	나는 개인적인 문제를 처리하는 능력에 대해 자신감을 느꼈다. (Confident about your ability to handle your personal problems)	0	1	2	3	4
8(7)	나는 일이 나의 뜻대로 진행된다고 느꼈다.(Things are going your way)	0	1	2	3	4
9(10)	나는 매사를 잘 컨트롤한다고 느꼈다. (Felt that you were on top of things)	0	1	2	3	4
10(11)	나는 통제할 수 없는 범위에서 일어난 일 때문에 화가 났다. (Anger because of things that happened that are outside of your control)	0	1	2	3	4

Park JO, Seo YS. Validation of the perceived stress scale (PSS) on samples of Korean university students. Korean J Psychol. 2010;29(3):611-29.

본 PSS 한국어 버전은 "박준호, 서영석 (2010). 대학생을 대상으로 한 한국판 지각된 스트레스 척도 타당화 연구. 한국심리학회지: 일반, 29(3), 611-629"에서 발췌한 것입니다.
